# Cultivation of *Anaplasma ovis* in the HL-60 human promyelocytic leukemia cell line

**DOI:** 10.1038/emi.2017.70

**Published:** 2017-09-20

**Authors:** Ran Wei, Hong-Bo Liu, Frans Jongejan, Bao-Gui Jiang, Qiao-Cheng Chang, Xue Fu, Jia-Fu Jiang, Na Jia, Wu-Chun Cao

**Affiliations:** 1 State Key Laboratory of Pathogen and Biosecurity, Beijing Institute of Microbiology and Epidemiology, Beijing 100071, China; 2 Utrecht Centre for Tick-borne Diseases (UCTD), Faculty of Veterinary Medicine, Utrecht University, Yalelaan 1, Utrecht 3584 CL, The Netherlands; 3 Department of Veterinary Tropical Diseases, Faculty of Veterinary Science, University of Pretoria, Private Bag X04, Onderstepoort 0110, South Africa

**Keywords:** *Anaplasma ovis*, HL-60 cell line, *in vitro* culture

## Abstract

The tick-borne bacterium *Anaplasma ovis* is a widely distributed pathogen affecting sheep, goats and wild ruminants. Here, the HL-60 human promyelocytic leukemia cell line was used to isolate *A. ovis* from PCR-positive sheep and goats in Heilongjiang Province, China. Two weeks after inoculation, morulae were observed in cytoplasmic vacuoles in four different HL-60 cultures. Confocal microscopy using a Cy3-labeled *A. ovis-*specific probe confirmed that the HL-60 cells were infected with *A. ovis*. Cells from the 6th HL-60 subculture displayed positive fluorescence when incubated with *A. ovis* antiserum in the indirect fluorescent antibody assay. PCR amplification and sequencing of 16S rRNA, *groEL*, *gltA*, *msp2* and *msp4 Anaplasma* genes revealed that the four *A. ovis* culture isolates were identical. Phylogenetic analysis showed that the sequences clustered with other *A. ovis* strains but could clearly be distinguished from other *Anaplasma* species. When the 18th subculture of infected HL-60 cells was examined by electron microscopy, lysosomes were often observed near the vacuoles. After the 24th subculture, Giemsa staining and PCR indicated that the HL-60 cells were negative for *A. ovis.* Although *A. ovis* can infect HL-60 cells for only four months, the ability of the organism to infect and multiply in HL-60 cells provides a tool to study intra-erythrocytic *Anaplasma* and host cell interactions.

## Introduction

*Anaplasma ovis* is an intra-erythrocytic tick-borne bacterial pathogen which mainly affects domestic goats and sheep,^1^ but has also been reported to be present in deer,^2^ wild boar ^3^ and domestic dogs.^4^ *A. ovis* infections are widely distributed and have been reported in North America, Europe and Asia.^5^ The organism can cause severe anemia, fever, weight loss, spontaneous abortion, jaundice and mortality in affected sheep or goats, thus resulting in economic losses in many countries.^6^ In 2007, the first human case of *A. ovis*, characterized by fever, hepatosplenomegaly and lymphadenopathy, was reported in Cyprus.^7^

*A. ovis* transmission is not well understood. The acquisition and transmission of *A. ovis* through different developmental stages of the various tick vectors have not been documented.^8^ It has been reported that *Rhipicephalus bursa* and *Dermacentor marginatus* are the potential vector ticks for *A. ovis* in Europe and North America, respectively.^9^ In Asia, in particular in China, *D. nuttalli*, *Hyalomma asiaticum* and *R. pumilio* are the potential vectors.^8^

Cultivation of *A. ovis* was attempted in transplantable cell lines in 1966, but these cell lines were not easily prepared.^10^ Similarly to *A. ovis*, *A. marginale* is another intra-erythrocytic bacterium and was successfully been cultivated in tick cell lines (IDE8 and BME26).^11, 12, 13^ These reports have provided good examples for studying *A. ovis*. However, tick cell lines are not easily managed and are not widely used in laboratories. We reasoned that cultivation of *A. ovis* in the HL-60 human promyelocytic leukemia cell line might be feasible, owing to the reported susceptibility of humans to infection with *A. ovis*. Here, HL-60 cells were infected with several isolates of *A. ovis* derived from infected sheep and goats from different herds, which acquired the infection in Heilongjiang Province in northeastern China.

## Materials and methods

### Infected blood sample preparation

EDTA blood samples were collected from goats and sheep in five counties in Mudanjiang City, Heilongjiang Province, northeastern China between May and August 2015. Subsequently, the blood samples were stored in liquid nitrogen with 10% dimethylsulfoxide, as a cryopreservant. The samples were tested and inoculated individually by using the following procedure: DNA was extracted from thawed blood samples with a QIAamp DNA Blood Mini Kit (QIAGEN, Germantown, MD, USA). Nested PCR reactions targeting the citrate synthase gene (*gltA*) of *A. ovis* were performed on all samples (Table 1). PCR products were sequenced to confirm the presence of *A. ovis* DNA. ‘*A. capra*’ was also assayed to exclude any co-infected samples by using a previously reported method.^14^ The sheep and goat sampling was performed in accordance with experimental animal administration and the ethics committee of the Academy of Military of Medical Sciences.

### Infection and monitoring of HL-60 cells

*A. ovis*-infected blood samples were inoculated into HL-60 cells (ATCC CCL-240) by using previously reported methods.^14^ Briefly, after the infected blood was quickly thawed and washed, 100 μL blood was added to a HL-60 cell culture with a density of 5 × 10^5^cells/mL. Cultures were maintained in RPMI 1640 medium (Gibco, Grand Island, NY, USA) with 10% heat-inactivated fetal bovine serum (Gibco, Grand Island, NY, USA) and 2 mM L-glutamine (HyClone, Logan, UT, USA) in a 37 °C incubator with 5% CO_2_. Cultures were examined twice per week. To maintain a density of (2–5) × 10^5^ cells per mL, fresh medium or non-infected HL-60 cells were added as required. Two weeks after inoculation, Wright-Giemsa staining was used to detect intracellular morulae in cytospins by light microscopy. After the infection was confirmed, the cell density was also maintained at (2–5) × 10^5^ cells/mL. Wright-Giemsa staining and PCR were performed weekly to monitor the infection.

### Combined Wright-Giemsa staining and fluorescence *in situ* hybridization

To identify the specificity of intracellular morulae observed in HL-60 cells, we developed an assay combining Wright-Giemsa staining with fluorescence *in situ* hybridization (FISH) to observe the same cytospin slide with two methods.

Cytospins were fixed in methanol and acetone (1:1, vol/vol) for 10 min and then fixed in methanol and acetic acid (4:1, vol/vol) for 15 min. First, FISH was performed with a commercial kit according to the manufacturer’s instructions (RIBOBIO, Guangzhou, China) with some modifications based on a patent description to improve the hybridization of the probe specifically to the bacterial and not the host cells.^15^ The FISH probes were designed specifically for *A. ovis* (RIBOBIO, Guangzhou, China). To achieve a sufficient signal-to-background ratio, multiple probes were targeted along each individual lncRNA/mRNA sequence of the *A. ovis msp*4 gene (KX579070). A set of 15–20 probes covering the entire length of the RNA molecule allowed for optimal signal strength, whereby each probe carried multiple fluorophore signals. A non-specific probe was also synthesized and included as a negative control. The probes were labeled with fluorescein isothiocyanate (FITC). The pooled FISH probes were re-suspended in a final concentration of 25 μM in RNase-free storage buffer and protected from light by storage at −20 °C.

After examination of FISH results under a microscope with a fluorescent light source, the same cytospin slide was washed with wash buffer for 3 min, followed by PBS for 3 min and Wright-Giemsa B solution for 3 min. Then, the same slide was stained by Wright-Giemsa as previously reported.^12^

### Confocal laser scanning microscopy

Cytospin slides prepared from the same infected culture as in FISH and Wright-Giemsa staining were used. The slides were fixed with methyl hydrate and acetone (1:1, vol/vol). The following process was similar to the aforementioned FISH assay, except that for confocal laser scanning microscopy, cyanine 3 (Cy3) labeled probes were used, and DAPI was used to counterstain the cell nuclei. In each experiment, a non-specific probe was used as a negative control. An Olympus Fluoview FV1000 automated inverted research microscope was used to observe the result. Images were acquired by using Olympus Fluoview Ver4.0b Viewer software in combination with a × 60 oil immersion objective lens and a numerical aperture of 1.25. The excitation wavelengths used were 405 nm (blue) and 594 nm (red). All experiments were conducted in triplicate.

### Electron microscopy

A 6th subculture and an 18th subculture of infected HL-60 cells and normal HL-60 cells, respectively, were processed, as previously described ^14^ for transmission electronmicroscopic examination using a CM-120 electronmicroscope (Philips Medical Systems, Cleveland, OH, USA).

### PCR, sequencing and phylogenetic analyses

Total DNA was extracted by using a QIAamp DNA Blood Mini Kit. Full length 16S ribosomal RNA gene (1461 bp) and partial sequences of *groEL*(1365 bp), *gltA* (804 bp), *msp2* (350 bp) and *msp4* (845 bp) were amplified with the primers and PCR conditions presented in Table 1. The sequences obtained were compared with previously published sequences deposited in GenBank by using BLAST (http://blast.ncbi.nim.nih.gov/Blast.cgi). Phylogenetic analyses were performed, and phylogenetic trees were constructed by using Mega 5.0 software.^16^

### Indirect Fluorescent Antibody assay

A 6th subculture of infected HL-60 cells was processed for the preparation of antigen slides. During the current survey on anaplasmosis, serum samples from goats and sheep were collected. The *A. ovis*-specific PCR-positive samples were used as positive controls, and another *Anaplasma* species infection was excluded by PCR targeting the 16S rRNA gene of the Anaplasmataceae family.^14^ *A. phagocytophilum* and *A. capra* infections were further excluded by using indirect fluorescent antibody assay to detect serum antibodies with a commercial kit (Focus Diagnostics, Cypress, CA, USA) or with our prepared *A. capra* antigen slides.^14^ Uninfected cells were stained with positive goat serum as a negative control to assess the background.

## Results

**Figure 1:**
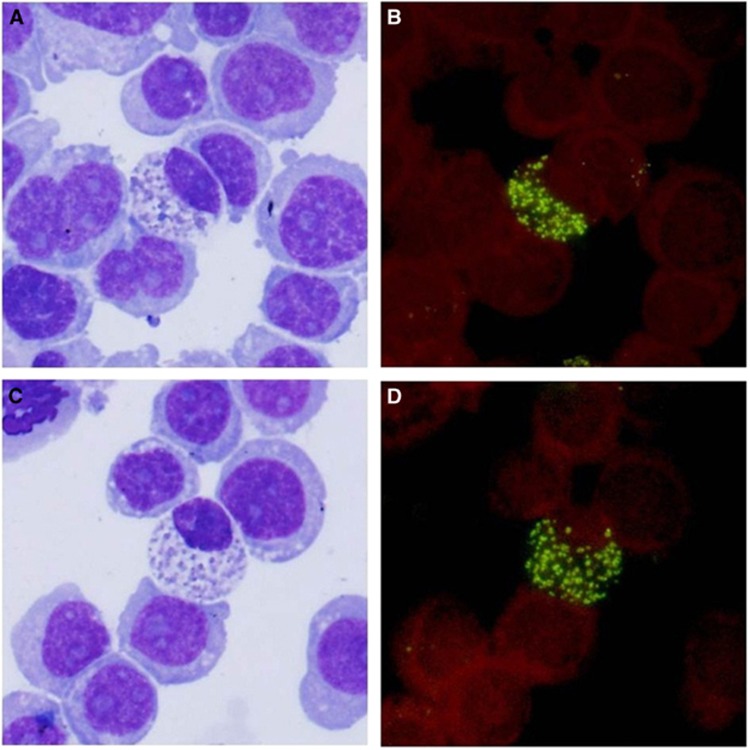
(**A** and **C**) Wright-Giemsa-stained cytospin of *Anaplasma ovis* in infected HL-60 cells. (**B** and **D**) Fluorescence insitu hybridization on the cytospin of the same HL-60 infected cells corresponding to (**A** and **C**). The probe was labeled with FITC.

Six PCR-positive *Anaplasma ovis* blood samples obtained from sheep and seven obtained from goats were inoculated into different HL-60 cell cultures, thus yielding four *Anaplasma ovis* isolates (two derived from sheep and another two from goats). Two weeks after the blood was inoculated, typical morulae were observed in the cytoplasm in the host cells. Wright-Giemsa-stained cytospins prepared 30 days post-inoculation from the 6th HL-60 subculture showed numerous small inclusion bodies in the cytoplasm (Figures 1A–1C). The same cytospin preparations derived from the 6th subculture were also examined by FISH. The intracellular localization of the fluorescence correlated well with microscope observations with Wright-Giemsa staining (Figure 1). The cells with non-specific signals in negative controls (either a non-specific probe reacting in *A. ovis*-infected cells or a specific probe in normal HL-60 cells) exhibited no staining with Wright-Giemsa stain (Supplementary Figure S1).

**Figure 2:**
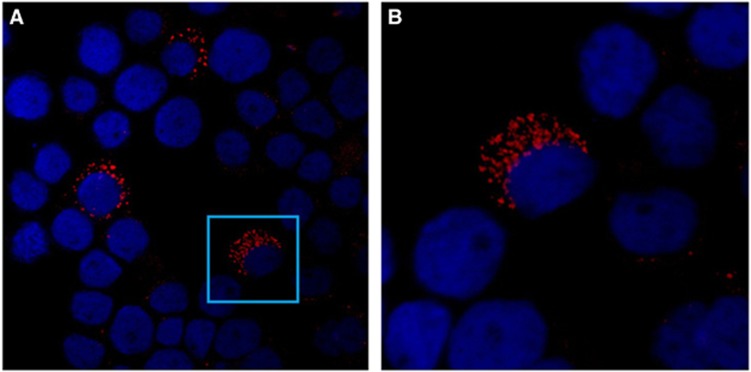
(**A**) Confocal laser scanner microscopy image showing three HL-60 cells infected with *A. ovis*. (**B**) The **A** inset shows one enlarged, positive HL-60 cell. The probe was labeled with Cy3.

**Figure 3:**
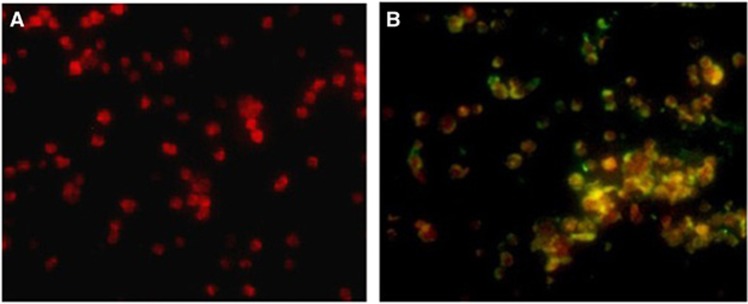
(**A**) Indirect fluorescent antibody assay image of uninfected HL-60 cells incubated with positive goat serum. (**B**) *Anaplasma ovis* in HL-60 cells detected by indirect fluorescent antibody assay using positive goat serum.

Confocal laser scanner microscopy confirmed that the positive fluorescence was actually due to the *A. ovis-*specific probe, and specific signals were homogeneously distributed within the cytoplasm of the HL-60 cell (Figure 2). The non-specific probe and non-infected HL-60 cells were used as controls, for which no specific reactions were observed. When cells from the 6th HL-60 subculture were used as an antigen in the indirect fluorescent antibody assay, the positive fluorescence obtained with the *A. ovis* antiserum confirmed that the cultures contained *A. ovis* (Figure 3).

**Figure 4:**
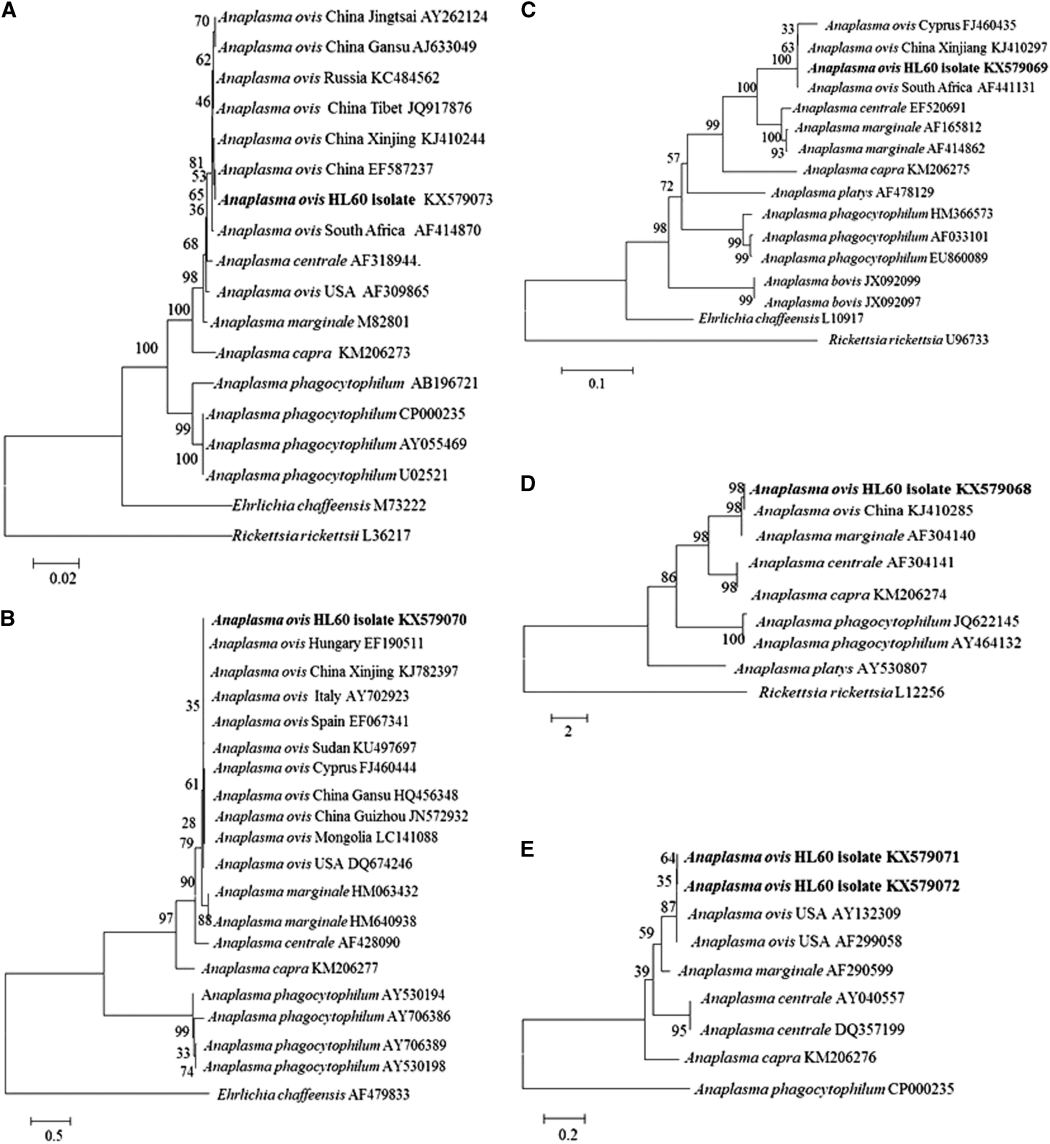
Phylogenetic analysis of *A. ovis* and other members of the family Anaplasmataceae. (**A**) is based on the 16S rRNA gene, (**B**) the *msp4* gene, (**C**) the *groEL* gene, (**D**) the *gltA* gene, and (**E**) the *msp2* gene. Phylogenetic analysis was conducted using maximum likelihood with the Kimura two-parameter plus γ rate model of substitutions. A bootstrap analysis of 1000 replicates was conducted to confirm the reliability of phylogenetic trees. The scale bar indicates the estimated evolutionary distance. GenBank accession numbers are provided after each isolate name.

Approximately 30 days post-inoculation and after six subcultures, there were no red blood cells remaining. PCR amplification of DNA derived from infected cells of the sixth subculture and subsequent sequencing of the 16S rRNA, *groEL*, *gltA*, *msp2* and *msp4 Anaplasma* genes revealed that the four *A. ovis* culture isolates were identical. Phylogenetic analysis clustered our sequences with other *A. ovis* strains, but clearly distinguished them from other *Anaplasma* species (Figure 4).

**Figure 5:**
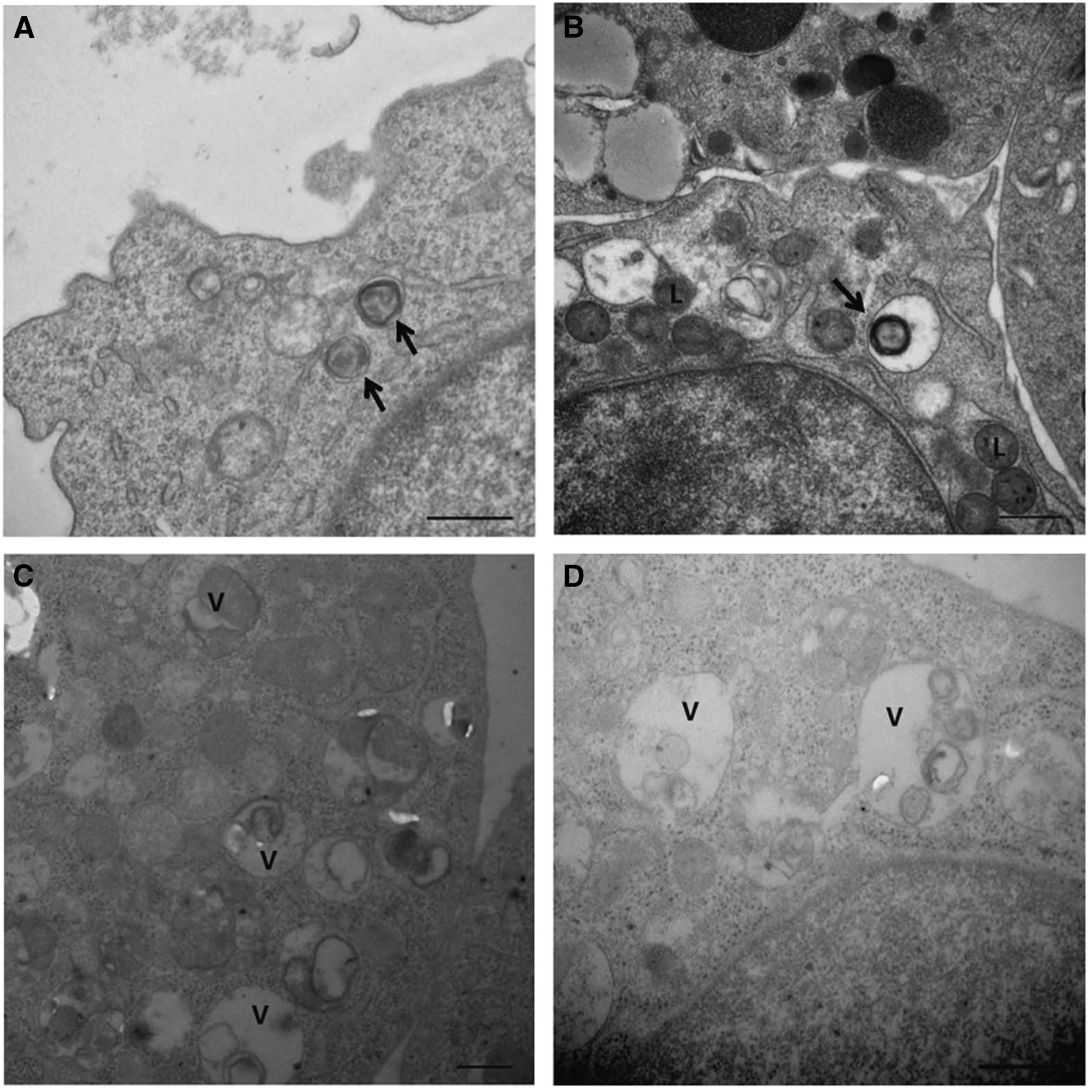
Electronphotomicrograph of HL-60 cells infected with *A. ovis*. (**A** and **B**) shows the 6th subculture. (**C** and **D**) shows the 18th subculture. *Anaplasma* organisms are indicated by the arrows. ‘L’ represents the lysosomal enzymes, and ‘V’ represents vacuoles containing cellular debris and amorphous material. The bars represent 500 nm.

Samples from the 6th and 18th subcultures of infected HL-60 cells were examined via electron microscopy. Intact morulae were seen in the cytoplasm of only a few cells in the 6th subculture (Figure 5A). Lysosomes were commonly observed in proximity to the vacuoles (Figure 5B). Numerous vacuoles containing cellular debris and amorphous material were observed in the 18th subculture (Figures 5C and 5D). After the 24th subculture, Giemsa staining and PCR indicated that the HL-60 cells were negative for *A. ovis*.

## Discussion

*Anaplasma ovis* is regarded as an important bacterial tick-borne pathogen affecting livestock. Although it can cause economic losses in animal husbandry and is a potential threat to human beings, there is a lack of information on the developmental biology of the organism and cellular invasion mechanisms. Therefore, our aim was to cultivate *A. ovis* by using the HL-60 promyelocytic leukemia cell line. Although *A. ovis* infected HL-60 cells, the infection could not be maintained beyond four months. The infection was confirmed by the observation of intracellular bacteria-specific FISH signals exactly corresponding to the Wright-Giemsa staining. The limited bacterial growth in infected cell cultures was confirmed by a positive PCR result for three months and five *A. ovis*-specific amplified gene sequences. The positive detection was not due to the remaining inoculated red blood cell or bacterial debris, because we regularly supplemented fresh medium or non-infected HL-60 cells into infected cultures to maintain the cell density.

HL-60 is derived from a patient with acute promyelocytic leukemia and can be induced by various agents to differentiate into granulocytes, monocytes or macrophages.^17^ We have previously used HL-60 to cultivate *A. capra*.^14^ Interestingly, HL-60 cells have been demonstrated to support the growth of *A. phagocytophilum*, as well as *Ehrlichia chaffeensis*.^18^ Although both *A. phagocytophilum* and *E. chaffeensis* are obligate intracellular pathogens, the former has a granulocytic tropism, and the latter has a monocyte-macrophage tropism.^19^ These results indicate that HL-60 cells possess a relatively broad potential to sustain infections with *Ehrlichia/Anaplasma* blood-dwelling organisms.

We noted that lysosomes were commonly seen in proximity to *A. ovis*-occupied vacuoles in HL-60 infected cells. HL-60 cells kill invading bacteria through oxygen-independent mechanisms, such as fusion of the phagosomes occupying bacterial cells with granules containing lysosomal hydrolytic enzymes.^20^ *A. phagocytophilum*, however, can be maintained in HL-60 cells because it is able to modulate vesicular trafficking and avoid clearance by the host cell.^21^ *A. phagocytophilum* resides in inclusions that are neither early nor late endosomes and does not fuse with lysosomes or Golgi-derived vesicles.^18^ We assume that the clearance of *A. ovis* by the infected HL-60 cells may be correlated with those accumulating lysosomes. It has been reported that both *A. marginale* and *A. phagocytophilum* interact with the endoplasmic reticulum via a complex series of events from within their respective pathogen-occupied vacuoles residing within the host cell cytoplasm.^22^ It has been postulated that similar interactions play roles in the maintenance of *A. ovis* within the respective pathogen-occupied vacuoles. Interestingly, there are no reports of *A. marginale* being supported by HL-60 cells, thus potentially indicating that other host cells are required for long-term maintenance of erythrocyte-borne *Anaplasma* species versus those species invading mainly host granulocytes.

The successful propagation of *A. marginale* and *A. odocoilei* sp. nov. (a new *Anaplasma* species from the white-tailed deer) in tick cell lines has promoted understanding of bacterial infectivity, cell tropism and vaccine development.^8, 23, 24, 25^ Our initial objective was to cultivate *A. ovis* in one easy-to-handle human cell line, owing to its potential to infect human beings. Although the *A. ovis* isolate in HL-60 cells was able to be established for only approximately four months, this cultivation can be used to prepare antigen slides to confirm any infection with this bacterium. This study does not clarify the clearance mechanism of *A. ovis* in HL-60 cells for the later passages. Through *in vitro* study of *A. ovis* transient infection in HL-60 cells and continuous infection in other cell lines to compare differences in cell responses, the effects of pathogen–host cell interactions on cell attachment, bacterial invasion and intracellular development of *A. ovis* can be explored.

## Figures and Tables

**Table 1:** PCR primers used in the study

**Gene**	**Primer pair**	**5′-3′ sequences**	**Annealing conditions**	**Amplicon size (bp)**
*16S rRNA*	Fd1	AGAGTTTGATCCTGGCTCAG	55	1461
	Rp2	ACGGCTACCTTGTTACGACTT		
	16SD	GGTACCYACAGAAGAAGTCC	52	
	16SR	TAGCACTCATCGTTTACAGC		
*groEL*	1048r	GGCTAGTCCTGCTGGTAAT	50	1365
	1474r	CGTTAGCGTAGTTCATGGTG		
	37f	AAATCTATAAGGGAGGTAGTGC	50	
	419f	AGGACGAAATTGCACAGG		
*msp4*	msp4f	GGGAGCTCCTATGAATTACAGAGAATTGTTTAC	60	845
	msp4r	CCGGATCCTTAGCTGAACAGGAATCTTGC		
*msp2*	67f	GCACCAGTCCATTCTTTG		350
	921r	ATCGGTCAGGAGGTCATA	50	
	418r	CGAACCTTTCATACCCTACT		
*gltA*	23f	GCGATTTTAGAGTGYGGAGATTG	53	804
	1104r	TACAATACCGGAGTAAAAGTCAA		
	148f	GGGTTCMTGTCYACTGCTGCGTG	53	
	940r	TTGGATCGTARTTCTTGTAGACC		
