# Supplementary material for: Cultivation of Anaplasma ovis in the HL-60 human promyelocytic leukemia cell line
Source: Emerg Microbes Infect. 2017 Sep 20;6(9):e83–. doi: 10.1038/emi.2017.70 (PMC5625320; doi:10.1038/emi.2017.70)

**Supplemental Figure S1** Panels **(A)** FISH on cytospin prepared from *A.ovis-*infected HL-60 cells with non-specific probe and **(B)** Wright-Giemsa-stained on the corresponding same cells. Panel **(C)** FISH on cytospin prepared from normal HL-60 cells with *A.ovis*-specific probe and **(D)**Wright-Giemsa-stained on the corresponding same cells.


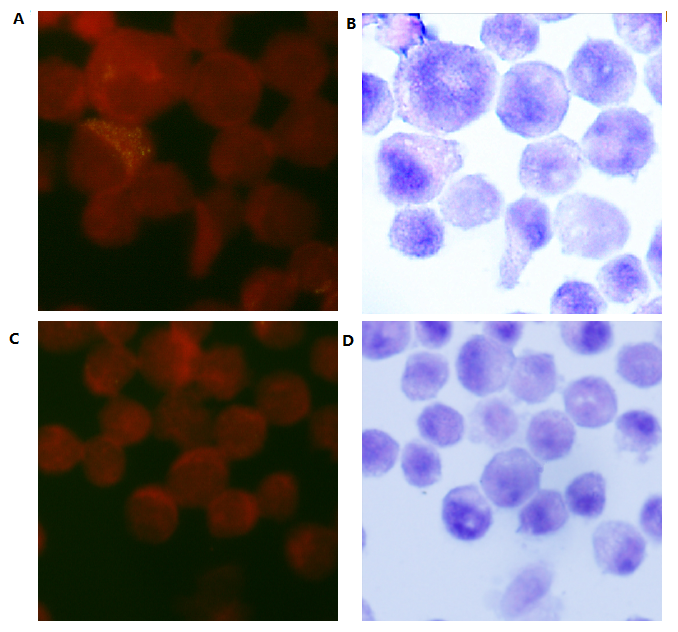

Supplement: Supplementary Figure S1 [file emi201770x1.docx]
